# Construction and validation of chronic pain prediction model after total knee arthroplasty

**DOI:** 10.12669/pjms.40.4.8979

**Published:** 2024

**Authors:** Juan Qian, Xuesong Wang

**Affiliations:** 1 Juan Qian, Department of Orthopedics, Affiliated Hospital of Jiangnan University, 1000 Hefeng Road, Wuxi City, Jiangsu Province 214000, China; 2 Xuesong Wang, Department of Orthopedics, Affiliated Hospital of Jiangnan University, 1000 Hefeng Road, Wuxi City, Jiangsu Province 214000, China

**Keywords:** Total knee arthroplasty, Chronic pain, Prediction model, Nomogram, Risk factor

## Abstract

**Objective:**

To explore the risk factors of chronic pain after total knee arthroplasty (TKA) and to establish and verify a prediction model.

**Methods:**

As a retrospective observational study, medical records of 239 patients who underwent TKA in Affiliated Hospital of Jiangnan University from January 2020 to December 2022 were reviewed. Fifty four patients suffered from chronic pain after TKA surgery. Univariate and multivariate logistic regression were used to analyze factors associated with the occurrence of chronic pain after TKA. A nomogram prediction model was established based on the identified independent risk factors, and its predictive effectiveness was evaluated.

**Results:**

Gender, postoperative 24-hourss numerical rating scale (NRS) and postoperative three months Hospital for Special Surgery Knee-Rating (HSS) scores were independent risk factors for chronic pain after TKA (*p*<0.05). The area of the receiver operating characteristic (ROC) of the nomogram model based on these factors was 0.904 (95% confidence interval [CI): 0.861-0.947), which indicates a good predictive value for the postoperative chronic pain. When the optimal cut off value was selected, the sensitivity and specificity of the model were 92.6% and 74.1%, respectively, indicating that the predictive model is effective.

**Conclusions:**

Gender, postoperative 24-hours NRS and postoperative three months HSS score are independent risk factors for chronic pain after TKA. The nomogram prediction model based on these factors is effective and can provide auxiliary reference for patients with chronic pain after TKA.

## INTRODUCTION

Total knee arthroplasty (TKA), one of the effective treatments for advanced arthritis of knee joint, can reliably relieve pain, correct joint deformities, and restore joint function..^1^,^2^ The number of TKA procedures is increasing worldwide due to recent advances in technology and gradually aging population.^2^,^3^

Chronic pain after TKA^4^ can be caused by postoperative infections, loosening of artificial joints, nerve compression or injury, soft tissue damage, and osteoporosis.^4^,^5^ In addition, muscle weakness, motor dysfunction, abnormal pain sensation and other conditions after surgery can also lead to chronic pain.^6^,^7^

Postoperative chronic pain can usually be controlled and alleviated through adequate rehabilitation training, drug treatment, and appropriate physical therapy.^8^ All these methods require close cooperation between the patient and the medical personnel.^8^,^9^ Early identification of patients who are at high risk of developing chronic pain after TKA and timely prevention will help improve their prognosis.^7^-^9^ To the best of our knowledge, only one study has constructed a nomogram prediction model for predicting chronic postoperative pain in elderly orthopedic patients.^10^ Therefore, this study aimed to explore the risk factors of chronic pain after TKA, and to construct a nomogram prediction model to verify the findings of previous study and provide guidance for clinical treatment.

## METHODS

As a retrospective observational study, medical records of 239 patients (132 males and 107 females) who underwent TKA surgery in Affiliated Hospital of Jiangnan University from January 2020 to December 2022 were reviewed.

### Ethical Approval

The study was approved by the institution’s Ethics Committee; Ref. No. 202031950 on January 10^th^ 2020.

### Inclusion criteria:

- Diagnosed as osteoarthritis and underwent initial TKA surgery.^11^
- The American Association of Anesthesiologists (ASA) grading is Level I and Level II.
- The clinical data were complete.

### Exclusion criteria:

- Emergency patients undergoing TKA surgery.
- History of hip or knee surgery or infection.
- Severe chronic pain at other sites before TKA surgery.
- Patients with an autoimmune disorder.
- Patients who were unable to complete study procedures due to cognitive impairment. .

### Outcome measures:

Preoperative anxiety and depression scores were evaluated by the Hamilton Anxiety (HAMA)^12^ and Depression (HAMD) scales,^13^ respectively. The HAMA scale consists of 14 items, with a total score of 56 points. The HAMD scale consists of 17 items, with a total score of 76 points. Higher score on both scales indicates more significant distress.

Pain status before and 24 hours after the surgery were measured by the Numerical Rating Scale (NRS)^14^ of 0-10, with 0 indicating no pain and 10 indicating severe pain.

Joint function was evaluated by the Hospital for Special Surgery Knee-Rating (HSS)^15^ knee joint function score before and 3 months after the surgery. The HSS score ranges from 0 to 100 points, and higher score indicates better knee joint function.

### Statistical Analysis

Statistical analysis was conducted using SPSS22.0 and R software version 4.0.0. The measurement data conforming to normal distribution were expressed as mean and standard deviation (SD), and the inter-group comparison was performed using independent sample *t* test. Non-normal distribution data were expressed by Median (interquartile range [IQR]), and Mann Whitney *U* test was used for comparison between the groups. Counting data were expressed by n (%), and Chi-squared test was used for comparison between groups. Logistic regression model was used to analyze the independent risk factors of chronic pain. Based on the identified risk factors, a nomogram prediction model was constructed using the “Rms” package in R software. ROC curve was established to analyze the prediction efficacy of the constructed model. Difference was considered statistically significant according to the test level of *p*<0.05.

## RESULTS

Age of the patients ranged from 50 to 90 years old, with an average of 70.67 ± 7.49 years. A total of 54 patients developed chronic pain after the operation. As summarized in Table-I, there was no significant difference between patients with chronic pain and patients without chronic pain in terms of education, marital status, occupational status, per capita monthly income of families, medical payment method, preoperative anxiety score, preoperative NRS score, preoperative HSS knee function score (*p*>0.05). Patients that experienced chronical pain were significantly older (72.61±5.46) compared to patients that did not suffer from pain (70.11±7.91), p=0.009. Female gender, high postoperative 24-hourss NRS, and low postoperative three months HSS were all significantly associated with chronic postoperative pain (*p*<0.05) (Table-I).

**Table-I:** Single factor analysis of postoperative chronic pain.

Index	No chronic pain (n=185)	Chronic pain (n=54)	χ^2^/t/Z	p
Gender (%)			7.534	0.006
Male	111(60)	21(38.89)		
Female	74(40)	33(61.11)		
Age (Years)	70.11±7.91	72.61±5.46	-2.652	0.009
Education level (%)			0.031	0.861
Below junior college	156(84.32)	45(83.33)		
College degree or above	29(15.68)	9(16.67)		
Marital status (%)			1.274	0.259
Married	159(85.95)	43(79.63)		
Others	26(14.05)	11(20.37)		
Per capita monthly income of households (yuan, %)			2.277	0.131
≤3000	124(67.03)	42(77.78)		
>3000	61(32.97)	12(22.22)		
Medical payment method (%)			0.630	0.427
Medical insurance	123(66.49)	39(72.22)		
Others	62(33.51)	15(27.78)		
Preoperative complications (%)			5.354	0.021
No	105(56.76)	21(38.89)		
Yes	80(43.24)	33(61.11)		
Preoperative anxiety score (points)	13(11,14)	12(10,14)	-1.404	0.16
Preoperative depression score (points)	12.25±3.57	11.44±3.19	1.49	0.138
Preoperative NRS score (points)	7(6,8)	7.5(7,8)	-0.033	0.974
Postoperative 24-hours NRS score	3(2,4)	5(5,6)	-8.642	<0.001
Preoperative HSS knee function score (points)	57.53±10.31	55.57±10.24	1.228	0.221
Postoperative three months HSS score	72(66,79)	67(62,72)	-3.525	<0.001

We then performed a logistic regression analysis using postoperative chronic pain as the dependent variable (0=No chronic pain group, 1=Chronic pain group) and gender, age, preoperative comorbidities, postoperative 24-hours NRS score, and postoperative three months HSS score” as covariates. The results showed that gender, postoperative 24-hours NRS score and postoperative three months HSS score were all independent risk factors of chronic pain after TKA (*p*<0.05) (Table-II).

**Table-II:** Binary Logistic Regression Analysis of chronic pain after TKA.

Variable	B	S.E.	Wald	p	OR	95%CI
Age	0.044	0.029	2.382	0.123	1.045	0.988~1.106
Gender	0.888	0.428	4.304	0.038	2.431	1.050~5.628
Preoperative complications	0.101	0.428	0.056	0.813	1.107	0.478~2.561
Postoperative 24-hours NRS score	1.439	0.222	42.077	<0.001	4.216	2.730~6.513
Postoperative three months HSS score	-0.077	0.027	7.893	0.005	0.926	0.878~0.977
Constant	-6.671	2.825	5.575	0.018	0.001	

**Fig.1:**
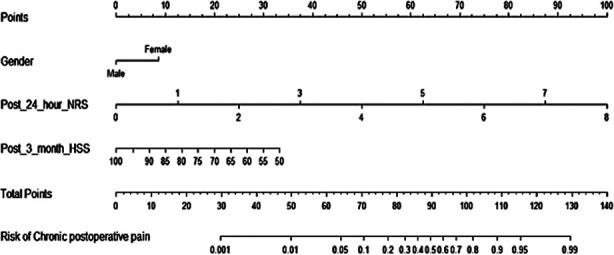
Nomogram prediction model.

**Fig.2:**
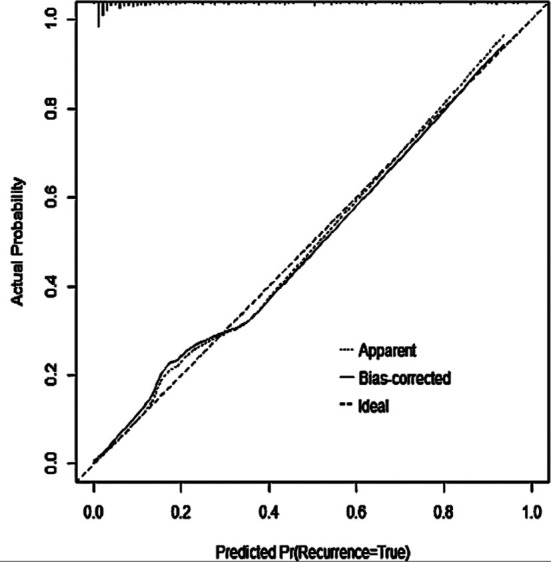
Calibration curve.

As shown in Fig.1, the nomogram predicted the postoperative chronic pain of patients. The C-index of the nomogram model was 0.904, indicating a good potential clinical effect of the prediction model. The calibration curve also showed good consistency between actual observations and nomogram predictions (Fig.2).

**Fig.3:**
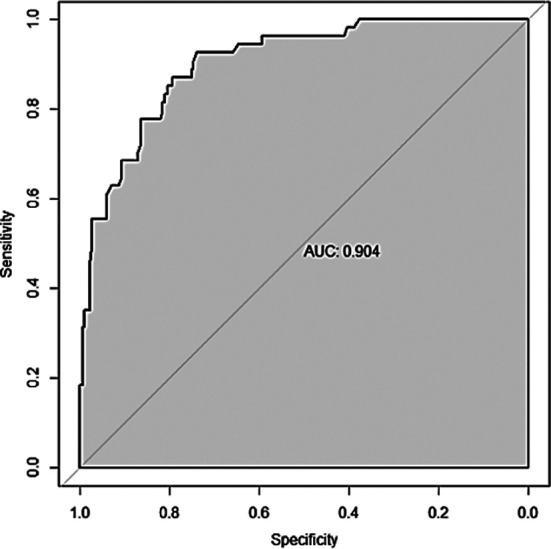
ROC of the model for predicting postoperative chronic pain.

ROC was established to analyze the value of this model in predicting postoperative chronic pain. The results showed that the area under curve (AUC) (95% CI) of this model was 0.904 (0.861-0.947), which has certain predictive value for postoperative chronic pain (Fig.3). When the optimal cut off value was selected, the sensitivity and specificity were 92.6% and 74.1%, respectively, indicating that the predictive model was effective.

## DISCUSSION

This study showed that female gender, high postoperative 24-hours NRS, and low postoperative three months HSS score were independent risk factors for chronic pain after TKA. The nomogram prediction model constructed based on the above factors has good predictive performance.

We speculate that women may be more prone to chronic pain due to specific physiological and psychological gender-specific characteristics.^16^,^17^ Pavlović et al.^18^ also showed differences in chronic pain predisposition between male and female patient. Female endocrine and nervous systems are more sensitive than males, and affected by various factors, which makes them more prone to chronic pain.^18^,^19^ In addition, hormonal changes during childbirth and menopause can affect pain sensitivity.^20^ Moreover, psychological and socioeconomic factors may also influence predisposition to chronic pain.^21^ Prego-Domínguez et al.^22^ showed in a cohort study of 16687 respondents that women with low socioeconomic status, high family burden and work pressure are more prone to chronic pain, which may also explain higher incidence of chronic pain in female patients after TKA in our study.^20^-^22^ Measures to reduce the risk of patients in developing chronic pain include regular postoperative rehabilitation training, reasonable analgesia program, and psychological support.^21^,^22^ In addition, personalized gender-specific treatment is also an effective way to reduce chronic pain.^23^

The postoperative 24-hours NRS can reflect a patient’s postoperative pain level directly. Early postoperative pain increases the risk of perioperative complications, affecting postoperative recovery and long-term pain persistence.^24^ Singh et al.^5^ studied the early pain response after TKA, with the follow up of six months after the operation in a cohort of patients (of them, 64.5% female). Their results showed that early postoperative pain may cause significant psychological discomfort,^5^ and lead to anxiety and fear, which may further exacerbate the patient’s pain perception.^5^,^24^ If this sense of pain persists, it may form a psychological memory and then produce a phenomenon similar to neuropathic pain, leading to long-term postoperative pain. A randomised controlled trial by Dawsey et al.^25^ showed that mindfulness training before total joint replacement can significantly improve postoperative pain and physical function. Furthermore, early postoperative pain may lead to muscle atrophy and dysfunction. If the patient cannot fully restore joint movement, it may lead to muscle atrophy and joint stiffness, which will eventually lead to long-term pain and dysfunction.^5^,^25^ Wylde et al.^26^ showed that local anesthesia infiltration has an impact on chronic postoperative pain after total hip replacement and knee joint replacement, and that regular postoperative rehabilitation exercises are necessary to help patients recover joint function and muscle strength as soon as possible, and alleviate long-term pain and dysfunction.

In addition, the study showed that low postoperative three months HSS score is one of the independent risk factors affecting chronic pain after TKA. Some studies have also shown that instability of the knee joint after TKA surgery can increase knee pain and movement limitations for six months after the surgery.^27^ This may be due to poor rehabilitation that may lead to muscle strength decline and joint stiffness that affect patient’s quality life, and increase pain perception.^28^ Additionally, postoperative complications may also lead to low HSS scores. For example, loosening or displacement of artificial joints, or infection may affect postoperative rehabilitation process, leading to ineffective recovery of joint function, and discomfort or pain.^29^ Moreover, factors such as obesity, old age, and severe bone and joint diseases can all slow postoperative recovery, affect joint function recovery, and increase the risk of postoperative pain.^30^ In order to reduce this risk, it is necessary to carry out timely rehabilitation exercises after the surgery, increase muscle strength and promote joint movement, to avoid problems such as joint stiffness and atrophy.^28^-^30^

Nomogram has been widely studied in predicting clinical outcomes after orthopedic surgery.^31^-^33^ The nomogram prediction model constructed based on the identified risk factors in the current study also showed a good prediction performance, which is consistent with the findings by Liu et al.^10^ The score of our nomogram model may be used to calculate the risk of postoperative chronic pain. The specified indicators are easy to obtain in clinical practice, which allows to perform early intervention. In addition, our study also verified the nomogram prediction model, and showed that it has a good prediction ability.

### Limitations

This was a single center retrospective study, which may affect the generalizability of our results. Moreover, the study population was in a small size, and the follow-up time was short. Prospective cohort studies are needed to further confirm our results. Our model needs to be evaluated in a broader population of patients with chronic pain after TKA.

## CONCLUSION

Gender, postoperative 24-hours NRS and postoperative three months HSS score are independent risk factors for chronic pain after TKA. The nomogram prediction model based on these factors is effective, and can provide auxiliary reference for patients with chronic pain after TKA.

### Authors’ contributions:

**JQ:** Conceived and designed the study.

**JQ** and **XW:** Collected the data and performed the analysis.

**JQ:** Was involved in the writing of the manuscript and is responsible for the integrity of the study.

All authors have read and approved the final manuscript.
